# Physiological Functions of the Cellular Prion Protein

**DOI:** 10.3389/fmolb.2017.00019

**Published:** 2017-04-06

**Authors:** Andrew R. Castle, Andrew C. Gill

**Affiliations:** Neurobiology Division, The Roslin Institute and Royal (Dick) School of Veterinary Sciences, University of EdinburghEdinburgh, UK

**Keywords:** prion, PrP^C^, transmissible spongiform encephalopathies, differentiation, adhesion, proliferation, myelin maintenance, stress protection

## Abstract

The prion protein, PrP^C^, is a small, cell-surface glycoprotein notable primarily for its critical role in pathogenesis of the neurodegenerative disorders known as prion diseases. A hallmark of prion diseases is the conversion of PrP^C^ into an abnormally folded isoform, which provides a template for further pathogenic conversion of PrP^C^, allowing disease to spread from cell to cell and, in some circumstances, to transfer to a new host. In addition to the putative neurotoxicity caused by the misfolded form(s), loss of normal PrP^C^ function could be an integral part of the neurodegenerative processes and, consequently, significant research efforts have been directed toward determining the physiological functions of PrP^C^. In this review, we first summarise important aspects of the biochemistry of PrP^C^ before moving on to address the current understanding of the various proposed functions of the protein, including details of the underlying molecular mechanisms potentially involved in these functions. Over years of study, PrP^C^ has been associated with a wide array of different cellular processes and many interacting partners have been suggested. However, recent studies have cast doubt on the previously well-established links between PrP^C^ and processes such as stress-protection, copper homeostasis and neuronal excitability. Instead, the functions best-supported by the current literature include regulation of myelin maintenance and of processes linked to cellular differentiation, including proliferation, adhesion, and control of cell morphology. Intriguing connections have also been made between PrP^C^ and the modulation of circadian rhythm, glucose homeostasis, immune function and cellular iron uptake, all of which warrant further investigation.

## The cellular prion protein and its gene

The cell surface glycoprotein known as the cellular prion protein (PrP^C^) has been the subject of intensive study since it was first proposed that misfolding of PrP^C^ plays a key role in the pathogenesis of the neurodegenerative disorders referred to as either the transmissible spongiform encephalopathies (TSEs) or prion diseases (Prusiner, 1982). Rather than focusing on the involvement of prion protein in disease, this review provides an overview of the physiological function of PrP^C^ as it is currently understood. However, before addressing PrP^C^ function, we first introduce some important aspects of the biochemistry/molecular biology of PrP^C^ and its gene (*PRNP*) that are of functional relevance, starting with an exploration of the evolutionary history of *PRNP*.

### Prion genes and their evolutionary history

**Figure 1:**
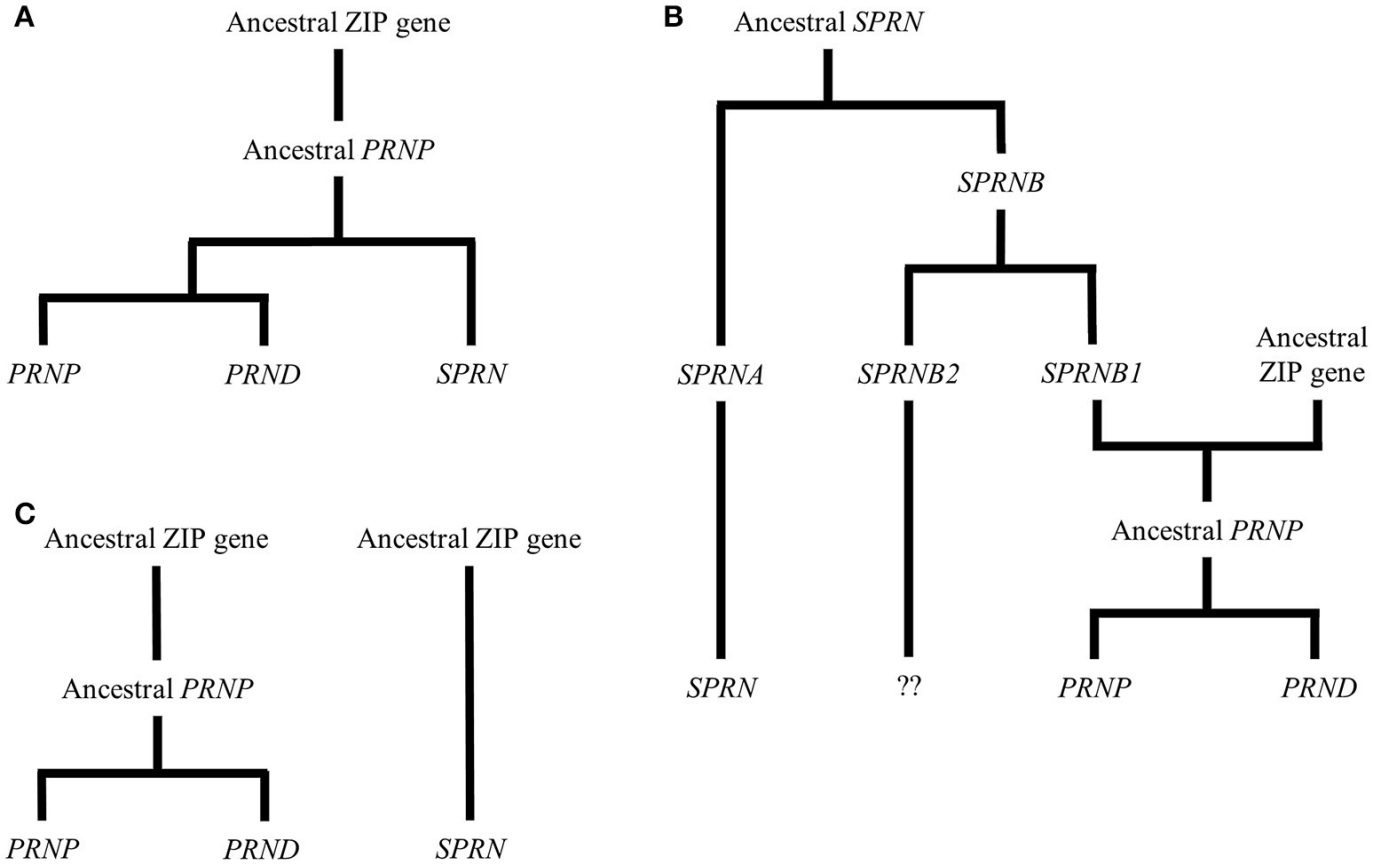
**Theories of the evolutionary history of the prion gene family**. The figure shows three possibilities for the evolution of the mammalian prion gene family. **(A)** Schmitt-Ulms et al. (2009) proposed that an ancestral *PRNP* gene evolved from a member of the ZIP metal ion transporter family. Subsequently, this *PRNP* precursor gave rise to the modern-day prion gene family through local duplications and other genomic rearrangements. **(B)** This alternative version, also put forward by Schmitt-Ulms et al. (2009), incorporates additional research by Premzl et al. (2004) suggesting that *SPRN* existed before *PRNP* and that the genetic material encoding the N-terminal domain of an ancestral PrP^C^ evolved from a gene called *SPRNB1* that, itself, had emerged from the original *SPRN*. The genetic material encoding the C-terminal domain of the ancestral PrP^C^ is proposed to have derived from a ZIP gene and a later, local duplication would have then created modern-day *PRNP* and *PRND*. Although, descendants of *SPRNB2* are found in fish, this gene is thought either to have been deleted or to have evolved beyond detectability in the mammalian lineage (Premzl et al., 2004). **(C)** A further possibility is that ancestral *PRNP* and *SRPN* genes could have evolved out of ZIP genes in separate events (Westaway et al., 2011).

**Figure 2:**
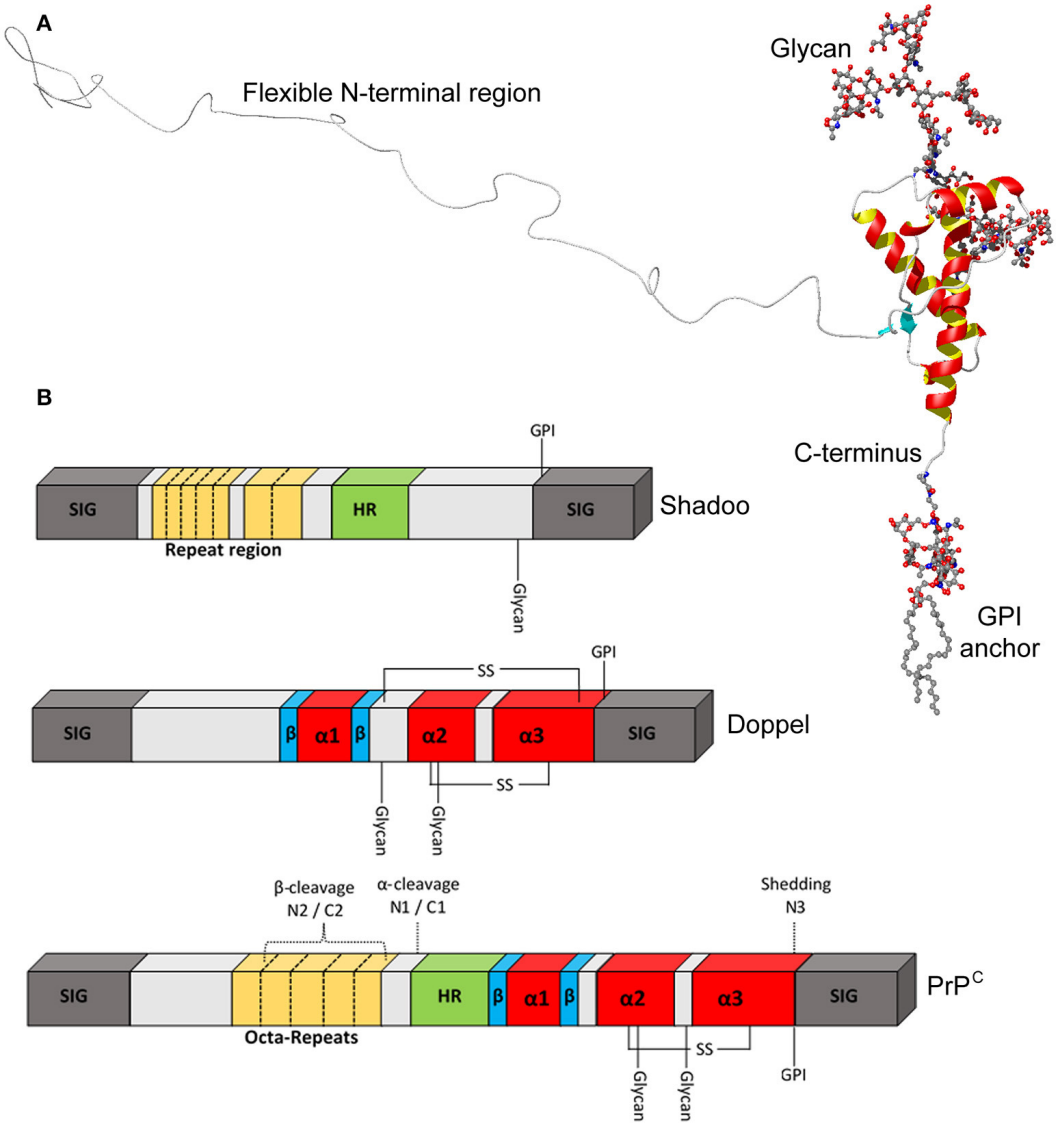
**Structural features of PrP^C^. (A)** Ribbon diagram of the PrP^C^ molecule. The C-terminal domain contains three α-helices, shown in red and yellow, and two β-strands shown in turquoise, whereas the N-terminal domain has been added on in a “random” configuration. **(B)** Schematic representation of PrP^C^, Sho, and Dpl to highlight key structural features in greater detail. PrP^C^ possesses octa-peptide repeats in the N-terminal region, whilst shadoo has two different, imperfect repeat stretches. SIG, N- and C-terminal signal peptides; HR, hydrophobic region.

PrP^C^ is encoded by the *PRNP* gene located on chromosome 20 (in humans—in mice this is chromosome 2) and is remarkably conserved throughout vertebrates. In addition to *PRNP*, the mammalian prion gene family includes *SPRN*, which encodes a protein referred to as shadoo (Sho), and *PRND*, which encodes a protein known as doppel (Dpl). Sho and Dpl share structural similarities to PrP^C^ (see below and **Figure 2**) and several theories have been put forward to explain the development of this gene family over evolutionary time. Three such theories are outlined in diagrammatic form in Figure 1. Figure 1A shows an explanation proposed by Schmitt-Ulms et al. (2009) and developed in other, more recent publications (Ehsani et al., 2011, 2012). The authors of these papers provided evidence that the prion gene family is evolutionarily descended from the LIV-1 branch of the ZIP (Zrt-, Irt-like) metal ion transporter family. Specifically, Ehsani et al. (2011) suggested that the ancestral *PRNP* gene could have been created as a result of reverse transcription of the mRNA of a ZIP gene, followed by insertion into the genome at a new position, a process known as retroposition. This event is proposed to have occurred around the time of the first vertebrates, which would explain the presence of *PRNP* homologs in all vertebrate lineages. In this model, a subsequent gene duplication event, in addition to some form of genomic rearrangement, led to modern-day *SPRN*, which is situated on chromosome 10 in the human genome. A later, local duplication would have then given rise to modern-day *PRNP* and *PRND*, which are directly adjacent to one another in the genome (Westaway et al., 2011). Figure 1B displays an alternative scenario also outlined by Schmitt-Ulms et al. (2009) but incorporating earlier research suggesting that *SPRN* may be evolutionarily more ancient than *PRNP* (Premzl et al., 2004). As per the first theory, the genetic material encoding the PrP^C^ C-terminal domain could have originated from an ancestral ZIP gene. Contrastingly, the sequences encoding the N-terminal domain may have arisen from a pre-existing *SPRN*-like gene (*SPRNB1*) that was created by earlier duplications of an *SPRN* founder gene. The generation of intergenic mRNA transcripts or an alternative form of local rearrangement could have caused these sequences to merge to form the ancestral *PRNP* (Westaway et al., 2011). A third model (Figure 1C) is that ancestral *PRNP* and *SRPN* genes could have evolved out of ZIP genes in separate events (Westaway et al., 2011).

### Regulation of *PRNP* expression

Although *PRNP* has a short GC-rich region immediately upstream of its transcription start site, as well as other features common to housekeeping genes (Puckett et al., 1991; Sakudo et al., 2010), intron 1 and the sequences upstream of the transcription start site also contain evolutionarily conserved, putative binding sites for numerous transcription factors, including Sp1 (Basler et al., 1986), activator proteins 1 and 2 (Mahal et al., 2001), forkhead box protein O3 (Liu et al., 2013), regulatory factor X1, heat shock factor 2, GATA-binding factor 3, thyrotrophic embryonic factor, myocyte enhancer factor 2, ecotropic viral integration site 1, E4 promoter-binding protein, 4 and nuclear matrix protein 4/cas-interacting zinc finger protein (Kim et al., 2008). These regulatory sequences presumably enable dynamic control of PrP^C^ expression in response to various stimuli, for example, treatment of cultured cells with nerve growth factor, insulin or insulin-like growth factor induces PrP^C^ expression (Kuwahara et al., 2000; Zawlik et al., 2006; Liu et al., 2013). Additionally, endoplasmic reticulum (ER) stress, oxidative stress and genotoxic stress are all reported to cause upregulation of PrP^C^ expression (Dery et al., 2013; Cichon and Brown, 2014; Bravard et al., 2015).

### Structure and basic intracellular trafficking of PrP^C^

PrP^C^ is synthesised as a precursor protein of 253 amino acids (human numbering) with an N-terminal signal peptide that codes for entry into the ER. However, seemingly because of an inefficiency in this ER-targeting signal (Rane et al., 2004), a small percentage of precursor molecules may fail to translocate fully into the ER lumen. Some of this PrP^C^ is consequently retained in the cytoplasm. Interestingly, the levels of this immature, non-translocated PrP^C^ may be upregulated by ER stress (Orsi et al., 2006). Another form, known as ^Ctm^PrP, partially enters the ER—the hydrophobic domain of PrP^C^ (see below) acts as a transmembrane domain, leaving the C-terminal region within the ER lumen and the N-terminal region in the cytoplasm. Reports suggest that ^Ctm^PrP is retained either in the ER or the Golgi apparatus before eventual degradation by the proteasome (Stewart et al., 2001, 2005). A second transmembrane form of PrP^C^ called ^Ntm^PrP can be produced if the molecule inserts into the ER membrane in the opposite orientation to ^Ctm^PrP. However, ^Ntm^PrP has rarely been studied in any detail (Chakrabarti et al., 2009). These forms appear more related to disease than physiological PrP^C^ function and are not considered further here.

The vast majority of immature PrP^C^ molecules translocate properly into the ER, enabling cleavage of a C-terminal signal peptide and addition of a glycophosphatidylinositol (GPI) anchor. Removal of both signal sequences results in a mature protein of 208 amino acids (residues 23–230 of the precursor protein), the structure of which is shown in Figure 2. The N-terminal domain is traditionally viewed as intrinsically disordered, but may possess elements of stable structure (Gill et al., 2000; Blanch et al., 2004; Taubner et al., 2010) that could enable PrP^C^ to interact with multiple partners (Bakkebo et al., 2015). The N-terminal domain also contains four tandem repeats of a sequence of eight amino acids, a region of PrP^C^ that is missing in Dpl. These octapeptide repeats may be functionally significant since analysis of PrP^C^ sequences from 53 species showed excellent conservation (Kim et al., 2008). A hydrophobic section (approximately residues 112–133) spans the divide between the N- and C-terminal domains and may be involved in PrP^C^ dimerisation (Rambold et al., 2008; Beland and Roucou, 2013). The PrP^C^ C-terminal domain has a globular structure, consisting of three α-helices, two β-strands and interconnecting loops (Riek et al., 1996; Haire et al., 2004). There is also a disulphide bond between residues 179 and 214 (Zahn et al., 2000) and N-linked glycans can be added at residues 181 and 197—the vast majority of PrP^C^ is generally thought to be di-glycosylated, although this may not be the case in all tissue/cell types (Williams et al., 2004). Notably, the entire globular domain is missing in Sho, but this protein does possess degenerate peptide repeats in the N-terminal region.

After passing through the ER, PrP^C^ moves to the Golgi apparatus where the N-linked glycans mature and the protein is sorted for trafficking to the cell surface. Once there, the GPI anchor attaches PrP^C^ to the extra-cytoplasmic face of the cell membrane, specifically in microdomains called lipid rafts (Vey et al., 1996). Dpl and Sho also both have GPI anchors and are therefore similarly targeted to the cell surface (Silverman et al., 2000; Watts et al., 2007). Interestingly, not all PrP^C^ is found at the cell surface. The protein seems to be subject to cycles of internalisation followed by trafficking back to the cell membrane *via* recycling endosomes (Shyng et al., 1993; Magalhaes et al., 2002; Sunyach et al., 2003), a process that presumably exists to enable tight control of the cell surface pool of PrP^C^. Studies using a green fluorescent protein reporter system have suggested that this recycling process may also result in PrP^C^ expression within the Golgi apparatus (Lee et al., 2001; Magalhaes et al., 2002; Nikles et al., 2008). Furthermore, there are reports that PrP^C^ can be present in the nucleus (Gu et al., 2003; Morel et al., 2008; Besnier et al., 2015; Bravard et al., 2015) and in mitochondria (Hachiya et al., 2005; Satoh et al., 2005; Sorice et al., 2012; Faris et al., 2017). Regardless of the different subcellular locations that PrP^C^ can reside, the functional form is believed to be that present on the cell surface.

### Spatiotemporal distribution of PrP^C^ expression

**Figure 3:**
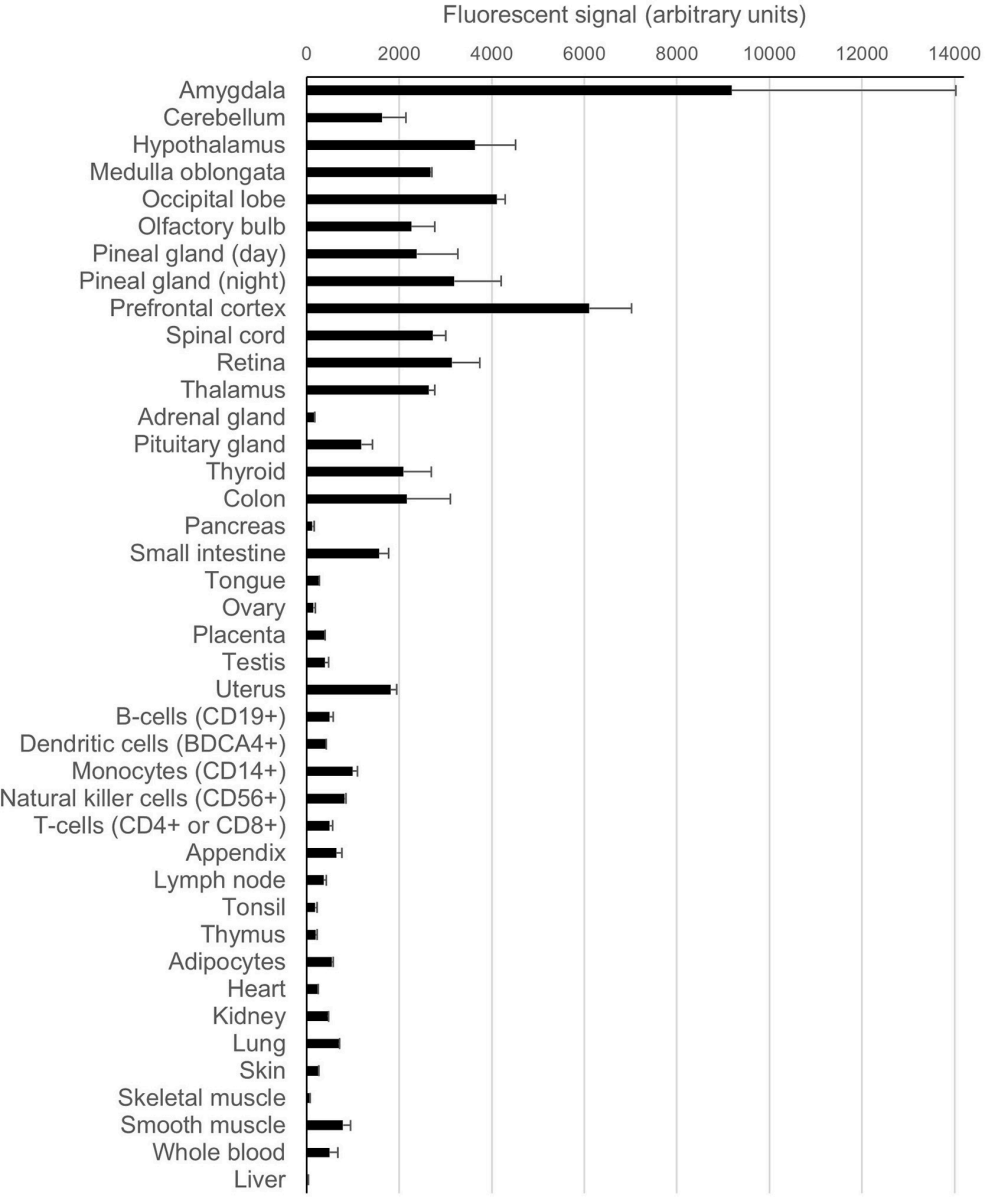
**Expression levels of *PRNP* in various human tissues and cell types**. The data for this chart originate from a publically available microarray dataset (GeneAtlas U133A, probeset 201300_s) originally published by Su et al. (2004) that was accessed through the BioGPS gene annotation portal (Wu et al., 2016). The bars on the chart indicate the arithmetic mean fluorescence signals from the replicate analytes; the error bars show standard error of the mean.

PrP^C^ is most highly expressed in the central nervous system (CNS) although *PRNP* transcripts can also be found in many other tissue/cell types, albeit at somewhat lower levels. Publically available *PRNP* expression data from microarray analyses of various human tissues and cell types (Su et al., 2004) are shown in Figure 3 in the form of a bar chart for purposes of comparison. By contrast, in the same microarray dataset *SPRN* transcripts were found to be restricted to CNS and peripheral nervous system (PNS) tissues, whilst *PRND* expression was confined largely to the testes. At the protein level, PrP^C^ expression in the brain appears to increase throughout development, reaching a peak in early life before reducing somewhat toward adulthood (Sales et al., 2002; Adle-Biassette et al., 2006). It is unclear how PrP^C^ levels in the brain are affected by the ageing process—one study found increased PrP^C^ expression in the brains of aged mice (Williams et al., 2004), whilst analysis of post-mortem human brain tissues showed that PrP^C^ expression in the hippocampus was significantly reduced in older individuals (Whitehouse et al., 2010).

Most reports indicate that PrP^C^ is expressed not only in neurons but also in astrocytes (Lima et al., 2007; Hartmann et al., 2013), oligodendrocytes (Moser et al., 1995; Bribian et al., 2012), and microglia (Adle-Biassette et al., 2006), although one study found that PrP^C^ expression was inhibited both *in vitro* and *in vivo* following differentiation of neural precursors into glial cell types (Steele et al., 2006). Non-neural cells of the forebrain also stain for PrP^C^, including the endothelial cells in blood vessel walls (Adle-Biassette et al., 2006). Furthermore, PrP^C^ expression has been reported in the PNS, including the dorsal and ventral root ganglia of the spinal cord (Tremblay et al., 2007; Peralta et al., 2012; Ganley et al., 2015), sensory and motor axons (Manson et al., 1992) and Schwann cells (Follet et al., 2002). Outside of the nervous system, PrP^C^ expression has been detected in immune cells, including T-lymphocytes, natural killer cells and mast cells (Durig et al., 2000; Haddon et al., 2009), and also in multiple organs, including the heart, pancreas, intestine, spleen, liver, and kidneys (Peralta and Eyestone, 2009).

### Proteolytic processing of PrP^C^

**Figure 4:**
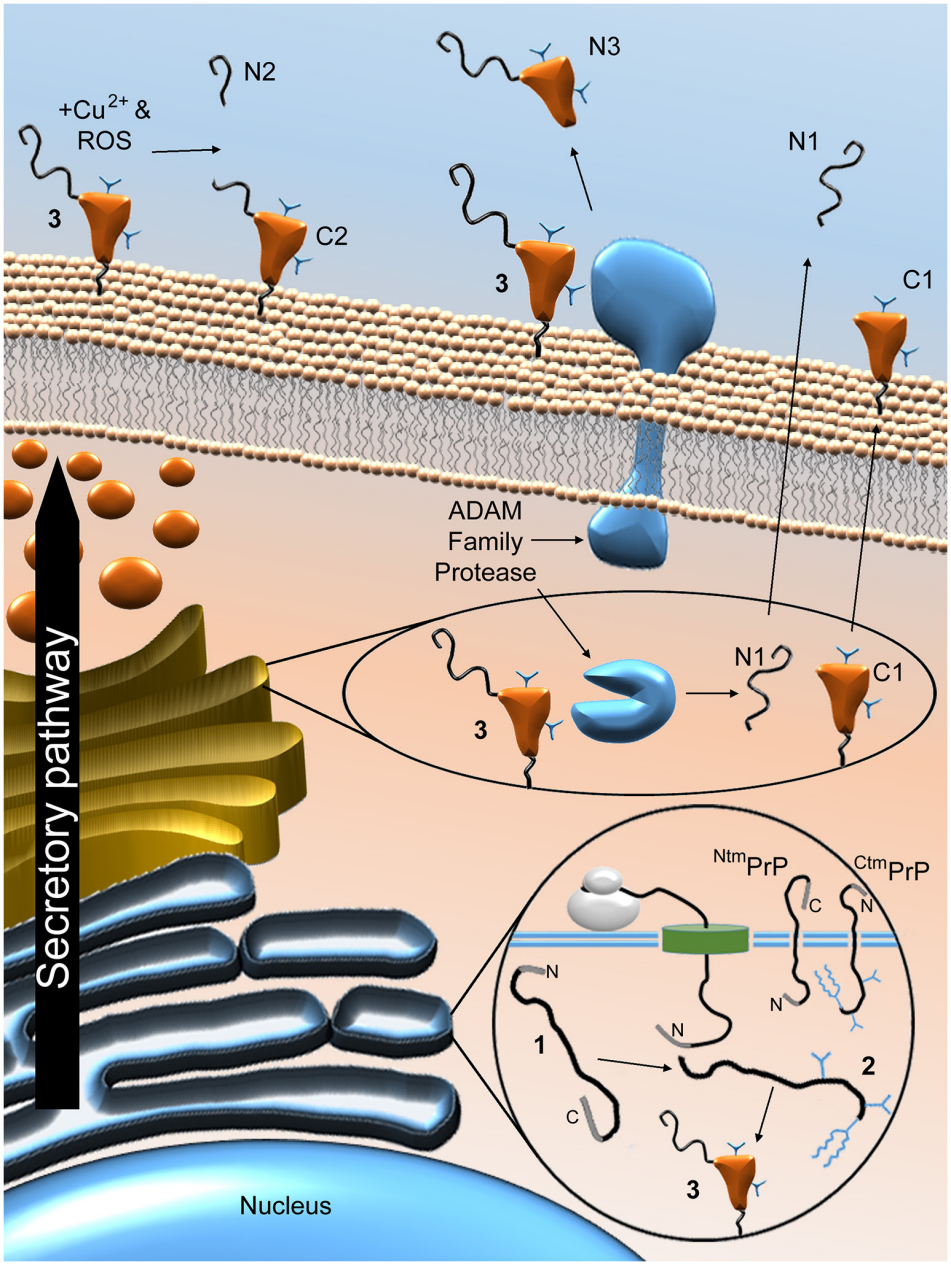
**Proteolytic processing of PrP^C^**. Post-translational and proteolytic processing events create multiple distinct PrP fragments. Ribosomal expression of PrP^C^ is concomitant with ER translocation. Imperfect translocation can result in ^Ntm^PrP or ^Ctm^PrP. Once in the ER, the immature protein (1) is N- and C-terminally truncated, glycosylated, the membrane anchor is added and the single disulphide bond is formed to produce the mature protein (2), before (potentially chaperone-mediated) folding to produce the folded form (3). Enzymatic α-cleavage, possibly mediated by ADAM family proteases, results in the production of N1 and C1 and is thought to occur either in an acidic endosomal compartment or within the Golgi apparatus. These fragments and the remaining, uncleaved PrP^C^ molecules are trafficked to the cell surface. Once there, PrP^C^ can be subject to β-cleavage, possibly stimulated by the combined presence of ROS and Cu^2+^, leading to the production of N2 and C2. ADAM protease-mediated shedding may also occur, which results in cleavage of PrP^C^ near its GPI anchor, thereby producing the N3 fragment. The sites of proteolytic cleavage are shown schematically in Figure 2B.

So far in this review, PrP^C^ has been referred to as a single entity. However, as summarised in Figure 4, PrP^C^ can be proteolytically processed in several ways. Firstly, PrP^C^ can be enzymatically cleaved at the peptidyl bond between residues 110 and 111 (human numbering) that lies just outside the hydrophobic domain of the protein (Chen et al., 1995; Watt et al., 2005). A recent study proposed that this “α” form of cleavage may also occur at alternative sites within the hydrophobic domain itself (McDonald et al., 2014). Members of the disintegrin and metalloproteinase domain-containing protein (ADAM) family may be responsible for α-cleavage (Vincent et al., 2001; Cisse et al., 2008; McDonald et al., 2014), although not all studies support this idea (Beland et al., 2012; Wik et al., 2012). α-Cleavage is thought to occur either in an acidic endosomal compartment (Shyng et al., 1993) or within the Golgi apparatus (Walmsley et al., 2009) and results in the production of the N-terminal fragment N1, which is released from the cell, and the C-terminal fragment C1, which seems to be trafficked to the cell membrane as per the full length protein (Harris et al., 1993; Vincent et al., 2000; Laffont-Proust et al., 2006). A large proportion of the cellular pool of PrP molecules may be in the form of C1—around 50% on average in sheep cerebral cortex, for example (Campbell et al., 2013). In addition to the α form of processing, PrP^C^ can be subject to cleavage within its octapeptide repeat region (McMahon et al., 2001). Recent findings from cell-free experiments using recombinant PrP suggest that this “β” cleavage can occur between the adjacent His and Gly residues of each octapeptide sequence (residues 61/62, 69/70, 77/78, and 85/86 for human PrP^C^) (McDonald et al., 2014). Whilst cleavage was equally likely at each site, the authors of this study caution that the presence of PrP^C^ binding partners may cause a particular site to be favoured *in vivo*. β-Cleavage seems to be dependent upon the combined presence of Cu^2+^ and reactive oxygen species (ROS) (McMahon et al., 2001), although enzymatic processing by ADAM8 is also a possibility (McDonald et al., 2014). The apparent role for ROS in β-cleavage suggests that it might be a response to oxidative stress (Watt et al., 2005), although β-cleavage also seems to occur physiologically, since the resulting C-terminal fragment, C2, is found in healthy brain tissues from various species, albeit in small amounts (Mange et al., 2004; Campbell et al., 2013). β-Cleavage is thought to take place at the cell surface, leading to retainment of C2 on the cell membrane and release of the N-terminal fragment N2 (Mange et al., 2004; Watt et al., 2005). In a third form of PrP^C^ processing, referred to as “shedding,” the protein can be cleaved at the 227/228 peptidyl bond (mouse numbering) by ADAM10, which removes the GPI anchor and the three adjacent amino acid residues, allowing the remaining protein fragment, sometimes known as N3, to be released into the extracellular medium (Taylor et al., 2009; McDonald et al., 2014).

### Why should we care about cellular prion protein function?

Before turning to PrP^C^ function, some introductory comments on the diseases that made the prion protein notorious are warranted, since the role that this protein plays in these diseases drives the abundance of studies into its function. Prion diseases are infectious diseases characterised by long, pre-clinical incubation periods followed by rapidly progressing neurodegeneration. Although rare, prion diseases are uniformly fatal and many mammalian species are susceptible (Fernandez-Borges et al., 2012). Animal prion diseases include scrapie in sheep and goats, bovine spongiform encephalopathy in cattle and goats, and chronic wasting disease in deer, whilst sporadic Creutzfeldt-Jakob disease is the most common human prion disease (Head, 2013). Early studies of the infectious agent suggested that it was too small and too resistant to UV irradiation to contain any living organism, even a virus (Alper et al., 1966, 1978; Alper, 1985). Subsequently, a protein was isolated from prion-infected hamster brains and its concentration appeared to be proportional to prion infectivity (Prusiner et al., 1982; McKinley et al., 1983). This discovery led to the prion hypothesis, which proposes that the novel protein is the major component of the infectious agent of all prion diseases, the term “prion” deriving from *pro*teinaceous *in*fectious particle (Prusiner, 1982). The infectious protein was later shown to be a host genome-encoded protein, now known as PrP^C^, that can undergo conversion to a partially protease-resistant, misfolded form referred to as PrP^Sc^ (McKinley et al., 1983; Prusiner et al., 1985; Horwich and Weissman, 1997). The misfolding process leads to a reduction in the amount of α-helical structure and an increase in β-sheet conformation (Pan et al., 1993), which makes PrP^Sc^ prone to aggregation. PrP^Sc^ is also thought to act as a template for further pathogenic conversion of the normally folded form (Horwich and Weissman, 1997), enabling disease to spread from cell to cell, from individual to individual and, in rarer instances, to transfer between species.

In addition to its role in prion disease transmission, PrP^Sc^ is thought to be involved in the disease processes that cause neuronal cell death, especially when aggregated into soluble oligomers (Ugalde et al., 2016). The loss of normal PrP^C^ function may also play a role in the pathogenesis of prion disease, since data from various animal models indicate that PrP^C^ levels fall as disease progresses (Mays et al., 2014). Given the absence of disease-modifying treatments for these diseases, therapeutic interventions that further reduce PrP^C^ expression or block its ability to interact with PrP^Sc^ are being investigated; better knowledge of PrP^C^ function would aid risk assessment of these approaches. Additionally, knowing which proteins interact with PrP^C^ physiologically might enable the development of other treatment strategies for prion diseases. Finally, as described later in this review, some beneficial functions of PrP^C^ could be exploited independently for therapeutic purposes. For these reasons, the physiological function of PrP^C^ has been investigated intensively over recent years and the knowledge gained from such research will be summarised in the following sections.

## Animal models for investigating PrP^C^ function

### PrP^C^-knockout mice

One way to assess protein function is to analyse the consequences of ablating expression in animals. As such, several groups have independently generated lines of PrP^C^-knockout mice, as summarised in Table 1. The first PrP^C^-knockouts were created in the early 1990s by gene targeting methods; these lines are referred to as Zurich I (Bueler et al., 1992) and Npu (Manson et al., 1994). Whilst the lack of PrP^C^ expression completely prevented scrapie transmission to these mice, thereby providing strong evidence for the prion hypothesis (Bueler et al., 1993; Prusiner et al., 1993), no other striking phenotypes were identified in initial analyses (Bueler et al., 1992; Manson et al., 1994). These results were surprising given that the structure and amino acid sequence of PrP^C^ are both well-conserved among mammalian species, which hints at an important function for the protein (Pastore and Zagari, 2007). However, more recent studies have identified several abnormalities of these PrP^C^-null mice and these findings will be covered in later sections.

**Table 1:** **PrP^C^-knockout mouse lines**.

**Knockout line**	**Year of 1st ref**.	**Genetic background**	**Ectopic Dpl?**	**Ataxia?**
Zurich I	1992	Mixed	No	No
Npu	1994	Pure (129/Ola)	No	No
Zurich III	2016	Pure (C57BL/6J)	No	No
Ki-Prnp-GFP	2008	Mixed	Yes (low level)	No
Rcm0	1995	Pure (129/Ola)	Yes (high level)	Yes
Ngsk	1996	Mixed	Yes (high level)	Yes
Rikn	2001	Mixed	Yes (high level)	Yes
Zurich II	2001	Mixed	Yes (high level)	Yes

In the years following the generation of the Zurich I and Npu PrP^C^-null mice, several groups independently produced additional knockout lines, known as Rcm0 (Moore et al., 1995), Ngsk (Sakaguchi et al., 1996), Rikn (Yokoyama et al., 2001), and Zurich II (Rossi et al., 2001). Unlike the Zurich I and Npu knockout mice, all the newer lines were shown to develop a late-onset ataxia due to death of cerebellar Purkinje neurons (Sakaguchi et al., 1996; Moore et al., 1999; Rossi et al., 2001; Yokoyama et al., 2001). The reintroduction of PrP^C^ into the Ngsk line rescued the ataxic phenotype, which seemed to confirm that it resulted from ablation of PrP^C^ expression (Nishida et al., 1999). However, the particular gene targeting methods used for production of the Rcm0, Ngsk, Rikn and Zurich II knockouts led to the generation of intergenic mRNA transcripts from the undisrupted, non-protein-coding exons of *Prnp* and the exons of the neighbouring *Prnd*. Therefore, Dpl was being expressed under the control of the *Prnp* regulatory sequences, leading to ectopic Dpl expression in the brains of the PrP^C^-null mice, but not the wild type controls (Moore et al., 1999). The presence of the ataxic phenotype only in knockout mice with ectopic Dpl expression suggested that the cerebellar Purkinje neurons were dying from Dpl-mediated neurotoxicity rather than a lack of PrP^C^. Further evidence obtained *in vitro* confirmed that Dpl was toxic to neuronal cells, but only in the absence of PrP^C^ expression (Sakudo et al., 2005; Qin et al., 2006). Although, the ability of PrP^C^ to block the neurotoxic effects of Dpl may derive from a physical interaction between the proteins (Qin et al., 2006), this is unlikely to be a genuine physiological function of PrP^C^ in the brain given that PrP^C^ and Dpl are not typically co-expressed.

The ectopic Dpl expression makes it difficult to interpret the phenotypes of RcmO, Ngsk, Rikn, and Zurich II PrP^C^-knockout mice. A further knockout line (Ki-Prnp-GFP), in which the protein-coding exon of *Prnp* was replaced with genetic material encoding enhanced green fluorescent protein (Heikenwalder et al., 2008), also exhibit aberrant expression of *Prnd* transcripts in the brain but the levels seem low enough not to cause any overt abnormalities (Jackson et al., 2014). However, one issue that does affect the Ki-Prnp-GFP line as well as the much more frequently studied Zurich I line is their mixed genetic backgrounds. As outlined in a review by Steele et al. (2007), the result is that the genomes of these PrP^C^-null mice and their wild type counterparts may differ at sites other than the *Prnp* locus. Importantly, even extensive backcrossing to one of the parental strains or to a different inbred strain is unlikely to eliminate this issue completely. This is because alleles of genes linked to *Prnp* that derive from the embryonic stem cells used for gene targeting will tend to persist in the PrP^C^-knockout line due to the low probability of recombination between neighbouring genes (Gerlai, 1996; Nuvolone et al., 2013). Therefore, rather than resulting from a lack of PrP^C^ expression, some of the phenotypes of the Zurich I PrP^C^-null mice may be due instead to the presence of different alleles of *Prnp*-linked genes compared with the wild type controls. Indeed, a specific phenotype associated with Zurich I (and Ngsk) PrP^C^-null mice—increased phagocytosis of apoptotic cells by macrophages—was traced to polymorphisms in the nearby gene that encodes signal-regulatory protein α-1 (Nuvolone et al., 2013). Due to their pure genetic backgrounds, the Npu line and a recently derived knockout line called Zurich III, which has been created using transcription activator-like effector nuclease genome editing technology (Nuvolone et al., 2016), are free from the problems caused by interfering linked genes.

### Other animal models for investigating PrP^C^ function

Although, mice have been used in the great majority of studies to investigate PrP^C^ function *in vivo*, PrP^C^ expression has also been knocked out in goats and in cattle, both natural hosts of prion disease. Whilst no in-depth phenotypic description of the PrP^C^-null goats has been published, the animals in question were reported to be healthy up to at least 5 months of age (Yu et al., 2009; Zhu et al., 2009). Similarly, detailed clinical and histopathological examinations in addition to analyses of blood samples and isolated peripheral blood lymphocytes uncovered no overt abnormalities of PrP^C^-null cattle (Richt et al., 2007). However, these observations were made only in relatively young individuals (up to 20 months of age) and the samples sizes were small, meaning that subtle phenotypes would have been difficult to detect.

PrP^C^ function has also been interrogated using zebrafish, in which three homologs of mammalian *PRNP* have been identified. These homologs are known as *prp1* and *prp2*, encoding the proteins PrP1 and PrP2, respectively, and a more divergent *PRNP*-like gene known as *prp3*, which codes for PrP3 (Cotto et al., 2005). Conflicting data on the spatiotemporal expression pattern of PrP1 during embryonic development have been published (Cotto et al., 2005; Malaga-Trillo et al., 2009). The evidence is clearer for PrP2 expression, which is reportedly absent in the early embryo but reaches high levels in the nervous system during later developmental stages and is maintained following hatching (Malaga-Trillo et al., 2009; Fleisch et al., 2013). Knockdown of PrP1 or PrP2 expression using morpholinos has identified putative roles for both proteins in regulating cell adhesion (Malaga-Trillo et al., 2009; Huc-Brandt et al., 2014; Sempou et al., 2016). Furthermore, complete knockout of PrP2 expression using zinc finger nuclease technology increased susceptibility to seizures (Fleisch et al., 2013), suggesting that PrP2 might modulate neuronal excitability. There is some evidence that mammalian PrP^C^ has similar functions to the zebrafish PrPs and this will be described in more detail in subsequent sections.

Recently, the first non-experimental animals without PrP^C^ expression were identified. Naturally occurring PrP^C^-null animals represent a useful resource for studying PrP^C^ function since they are free from the confounding factors that can be introduced by genetic manipulation. The animals in question were goats of the Norwegian Dairy Goat breed that were homozygous for a *Prnp* allele with a premature stop codon at position 32, just a few amino acids after the end of the N-terminal signal peptide (Benestad et al., 2012). These PrP^C^-null goats appeared to reproduce and behave normally (Benestad et al., 2012), although it has been reported that they may have increased red blood cell and neutrophil counts (Reiten et al., 2015). One would expect novel insights to arise from these animals in due course.

## PrP^C^ function

Significant research efforts have been directed toward investigating the physiological function of PrP^C^ in various cellular and animal models. The results from these studies have suggested roles for PrP^C^ in numerous processes, as will be explained in the following sections. In order to simplify our description of PrP^C^ function, we have aimed to group together putative functions that may be related, including some that may seem unconnected at first glance but could, in fact, result from PrP^C^ regulating the same underlying biochemical pathways.

### Stress-protection

The most extensive body of work relating to PrP^C^ function addresses the putative stress-protective properties of the protein. Initial evidence came from PrP^C^ protecting neuronal “HpL” cells from serum withdrawal (Kuwahara et al., 1999)—the loss of growth and survival factors present in serum results in activation of mitochondria-dependent apoptotic signalling driven by a protein called Bax (Deckwerth et al., 1996). Further studies using HpL cell lines appeared to confirm that PrP^C^ expression confers protection against serum deprivation-induced apoptosis (Kim et al., 2004; Wu et al., 2008). However, as described by Kuwahara et al. (1999), the HpL cell lines were generated by immortalisation of primary hippocampal neurons derived from embryonic Rikn PrP^C^-null mice. As explained previously, Dpl is ectopically expressed in the brains of these mice; consequently, the HpL cell lines also express Dpl (Sakudo et al., 2005). Therefore, the greater susceptibility of HpL cells to serum deprivation compared with control cell lines derived from wild type mice may have been caused by the toxic effects of Dpl rather than the absence of PrP^C^ expression. Additionally, the increased resistance to serum withdrawal following reintroduction of PrP^C^ into the HpL cells (Kim et al., 2004; Wu et al., 2008) may merely have been caused by PrP^C^ interacting with Dpl and inhibiting its neurotoxicity (Moore et al., 1999; Sakudo et al., 2005; Qin et al., 2006), which, as previously mentioned, is unlikely to be a physiologically relevant function of PrP^C^. More recently, the Zurich I line of PrP^C^-knockout mice, which do not have ectopic Dpl expression in the CNS, was used for the production of additional immortalised hippocampal cell lines, although there have been conflicting reports over whether or not PrP^C^ expression confers significant protection against serum deprivation in these cells (Nishimura et al., 2007; Oh et al., 2008). Nonetheless, PrP^C^ expression has been shown to protect human primary neurons and MCF-7 breast cancer cells from microinjection or transfection with Bax-expressing constructs (Bounhar et al., 2001; Roucou et al., 2005). In addition, viability of MDA-MB-435 breast cancer cells following serum deprivation was reduced by RNA interference-mediated knockdown of PrP^C^ expression (Yu et al., 2012).

A number of studies have assessed how PrP^C^ affects the cellular response to staurosporine, a potent but relatively non-selective adenosine triphosphate-competitive kinase inhibitor (Ruegg and Burgess, 1989) that seems to induce mitochondria-independent apoptotic signalling as well as affecting the same apoptotic pathways activated by serum deprivation (Zhang et al., 2004). PrP^C^ expression has been reported to protect primary hippocampal neurons from staurosporine-mediated cell death, possibly through an interaction with stress-induced phosphoprotein 1 (STI1) (Lopes et al., 2005; Beraldo et al., 2010; Ostapchenko et al., 2013). STI1 is a secreted protein thought to interact with PrP^C^, leading to activation of the pro-survival protein kinase A (PKA) signalling pathway (Zanata et al., 2002; Lopes et al., 2005). Interestingly, studies using primary cortical neurons derived from Zurich I PrP^C^-knockout mice found that transfection with a vector encoding full length PrP^C^ actually increased susceptibility to staurosporine (Paitel et al., 2004), whereas the N1 fragment produced by α-cleavage was neuroprotective (Guillot-Sestier et al., 2009). There are several additional reports of full length PrP^C^ expression conferring reduced viability in response to staurosporine exposure in a number of cell lines (Paitel et al., 2002, 2003; Sunyach et al., 2007; Guillot-Sestier et al., 2009).

In addition to direct effects on apoptosis, PrP^C^ reportedly protects cells from oxidative stress. For example, basal levels of ROS and lipid peroxidation were lower in PrP^C^-transfected neuroblastoma and epithelial cell lines compared with untransfected controls (Rachidi et al., 2003; Zeng et al., 2003). Moreover, PrP^C^ expression by primary neurons, astrocytes and cell lines has been associated with lower levels of damage following exposure to various oxidative toxins (Brown et al., 1997b, 2002; Anantharam et al., 2008; Dupiereux et al., 2008; Bertuchi et al., 2012). A possible mechanism is that PrP^C^ modulates the activities of the antioxidant enzymes that convert ROS into less toxic products—several studies have shown lower superoxide dismutase and glutathione peroxidase activities in the absence of PrP^C^ expression (Brown et al., 1997b; Miele et al., 2002; Rachidi et al., 2003; Sakudo et al., 2003; Paterson et al., 2008). Since ROS may induce β-cleavage of PrP^C^ in the presence of Cu^2+^, the C2 or N2 fragments could be responsible for the putative antioxidant properties of PrP^C^. Indeed, it has been reported that N2 lowers ROS production in response to serum deprivation in neuronal cell lines and neural stem cells (NSCs) (Haigh et al., 2015a,b). As well as potentially increasing the activities of antioxidant enzymes, a recent study proposed that PrP^C^ translocates to the nucleus in response to oxidative stress-induced DNA damage and directly activates the base excision repair pathway by interacting with AP endonuclease and enhancing its activity (Bravard et al., 2015). Additionally, PrP^C^-mediated activation of the tyrosine-protein kinase Fyn might lead to a stress-protective release of calcium ions from stores in the ER (Krebs et al., 2007). However, not all the evidence agrees with an antioxidant role for PrP^C^. Other studies have found no differences in superoxide dismutase activity between PrP^C^-null and wild type mice in the spinal cord, spleen or brain (Hutter et al., 2003; Steinacker et al., 2010). Additionally, although PrP^C^ protected neuroblastoma cells from the oxidative toxin 3-morpholinosynonimine hydrochloride through activation of the phosphatidylinositol-4,5-bisphosphate 3-kinase (PI3K)-RACα serine/threonine-protein kinase (Akt) signalling pathway, PrP^C^ transfection actually increased susceptibility to hydrogen peroxide treatment (Vassallo et al., 2005). Incidentally, PI3K-Akt signalling can also activate the mammalian target of rapamycin (mTOR) pathway, leading to reduced levels of autophagy, thereby rationalising the reported regulation of autophagic flux by PrP^C^ (Nah et al., 2013; Shin et al., 2014).

Finally, PrP^C^ has been implicated in the response to ER stress, which is caused by accumulation of unfolded/misfolded proteins within the ER. A cell responds to ER stress by triggering the unfolded protein response, which consists of: (1) increased expression of chaperones to improve protein folding; (2) global inhibition of protein synthesis; (3) an increase in ER volume; and (4) activation of the ER-associated protein degradation pathway, a key pathway in neurodegenerative disorders (Halliday and Mallucci, 2014). Interestingly, ER stress-response elements have been found within the human *PRNP* promoter and they appear active, since PrP^C^ expression was induced following treatment of breast carcinoma cells with toxins that cause ER stress (Dery et al., 2013). In the same study, knockdown of PrP^C^ expression in several cancer cell lines resulted in increased cell death in response to these toxins. Higher PrP^C^ expression in spite of the global, ER stress-induced reduction in protein synthesis suggests a potential role for PrP^C^ in the unfolded protein response, although results from other studies argue against a protective role for PrP^C^ during ER stress (Roucou et al., 2005; Anantharam et al., 2008).

In conclusion, the idea that PrP^C^ has a direct stress-protective function remains unproven. For example, there is no clear data supporting a protective role for PrP^C^ expression in the response to ER stress and, depending on the cellular context, PrP^C^ expression is seemingly either pro-survival or pro-apoptotic following exposure to staurosporine. There is evidence that PrP^C^ expression protects cells from serum deprivation, although this is weakened by the use of the Dpl-expressing HpL cells in several of the relevant studies. Arguably, the evidence that PrP^C^ can protect cells from oxidative stress is more convincing, although, as far as we are aware, this putative function has yet to be confirmed in the genetically pure Npu or Zurich III lines of PrP^C^-knockout mice.

### Cellular differentiation

In addition to its reported role in neuroprotection, several studies have shown that PrP^C^ promotes neurite outgrowth, and potential explanations include interactions of PrP^C^ with STI1 (Lopes et al., 2005), neural cell adhesion molecule 1 (NCAM1) (Santuccione et al., 2005), epidermal growth factor receptors (Llorens et al., 2013), integrins (Loubet et al., 2012), laminin (Graner et al., 2000a), or metabotropic glutamate receptors (mGluRs) (Beraldo et al., 2011). The downstream signalling responsible may include inhibition of the ras homolog gene family, member A (RhoA)-Rho-associated protein kinase (ROCK) pathway (Loubet et al., 2012). When activated, this signalling pathway stabilises the actin cytoskeleton, thereby inhibiting the development of filopodia—dynamic protrusions from the neurite growth cone that respond to the extracellular environment to guide migration of the developing neurite (O'Connor et al., 1990). Activation of the extracellular signal-regulated kinases 1 and 2 (ERK1/2), PI3K-Akt, and protein kinase c (PKC) signalling pathways may also be involved in mediating PrP^C^-dependent neurite outgrowth (Lopes et al., 2005; Caetano et al., 2008; Beraldo et al., 2011; Llorens et al., 2013). It should be cautioned that signal-regulatory protein α-1 reportedly modulates neurite outgrowth (Wang and Pfenninger, 2006) and, as previously mentioned, polymorphisms between Zurich I PrP^C^-null mice and their wild type counterparts in the gene encoding signal-regulatory protein α-1 can be present even after extensive backcrossing to an inbred strain (Nuvolone et al., 2016). This confounding factor affects the interpretation of several studies that used cells derived from these mice to show apparent regulation of neurite outgrowth by PrP^C^ (Lopes et al., 2005; Santuccione et al., 2005; Beraldo et al., 2011). However, investigations of other cell lines are free from this problem and a connection between PrP^C^ expression and neurite outgrowth has been demonstrated repeatedly in such models (Graner et al., 2000a,b; Loubet et al., 2012; Llorens et al., 2013).

Neurite outgrowth is a feature of neuronal differentiation, which raises the possibility that the effects of PrP^C^ expression on neurite outgrowth may be consequences of PrP^C^ regulating differentiation. Indeed, such a role may not be restricted to the nervous system, since there is evidence that PrP^C^ can influence some of the earliest differentiation processes that occur during embryogenesis. For example, PrP^C^ may indirectly modulate NCAM1 polysialylation in order to regulate the epithelial-to-mesenchymal transition (Mehrabian et al., 2015, 2016), a vital developmental process that alters cell adhesion and enables cell migration. Ectodermal cells in the developing embryo must undergo epithelial-to-mesenchymal transition as part of gastrulation, which may explain why knockdown of PrP1 expression in zebrafish embryos has been shown to prevent this process (Malaga-Trillo et al., 2009). This finding suggests that PrP^C^ may have an evolutionarily-conserved role in regulating cell adhesion. Indeed, zebrafish PrP1 and PrP2 are reported to regulate the connections between adherens junctions and the actin cytoskeleton, mainly by affecting the localisation of E-cadherin and β-catenin to these junctions (Malaga-Trillo et al., 2009; Sempou et al., 2016). This process seems to rely upon signalling through members of the Src family of tyrosine-protein kinases (Sempou et al., 2016) that could be mediated by PrP1/2 interacting with the zebrafish NCAM1 ortholog (Santuccione et al., 2005). PrP^C^ has been reported to regulate other differentiation processes. For example, knocking down PrP^C^ expression in cultured human embryonic stem cells delayed spontaneous differentiation into the three germ layers (Lee and Baskakov, 2013). In a similar manner, PrP^C^ expression has been shown to promote guided differentiation of cultured human embryonic stem cells and neural precursors into neurons, astrocytes and oligodendrocytes (Steele et al., 2006; Lee and Baskakov, 2014). Furthermore, a role in regulating cell adhesion suggests that PrP^C^ expression in cancer may affect the tendency to metastasise. Indeed, Chieng and Say (2015) found that overexpression of PrP^C^ in a colon adenocarcinoma cell line increased invasiveness, whilst a study of pancreatic ductal adenocarcinoma patients reported that PrP^C^-positive tumours were associated with reduced survival time from diagnosis (Sy et al., 2011). PrP^C^ expression was also shown to be upregulated in cancerous tissue from human gastric cancer patients compared with adjacent non-cancerous tissue from the same individuals (Zhou et al., 2014), although a recent study of a larger patient group found the opposite correlation (Tang et al., 2016).

Differentiation often involves a change in cell morphology, such as neurite extension, and this will likely require altered expression of cytoskeletal proteins. Several proteomic studies have identified such changes in cell lines in which PrP^C^ was knocked down or overexpressed (Provansal et al., 2010; Weiss et al., 2010; Mehrabian et al., 2014) and also in PrP^C^-null liver tissues compared with wild type controls (Arora et al., 2013). Analyses of brain tissues have been less successful at detecting differential expression of cytoskeletal proteins (or of any proteins for that matter), although this could be because cell type-specific effects of PrP^C^ are averaged out over an entire tissue (Crecelius et al., 2008; Mehrabian et al., 2016). Another common feature of differentiation is a change of cell cycle progression. For example, neural precursors withdraw from the cell cycle as they differentiate into post-mitotic neurons. In this regard, PrP^C^ has been shown to inhibit proliferation of oligodendrocyte precursors (Bribian et al., 2012), neuronal cells (Kim et al., 2005) and cells derived from the intestinal epithelium (Morel et al., 2008). In the case of NSCs, one study found that expression of full length PrP^C^ promoted proliferation, whilst the N1 and N2 fragments appeared to be inhibitory (Haigh and Collins, 2014). Interestingly, proliferation and self-renewal of NSCs and their differentiation into neurons seem to rely, in part, upon signalling mediated by ROS (Le Belle et al., 2011) and the effects of the different N-terminal PrP fragments on NSC proliferation apparently arose from their modulation of intracellular ROS levels (Haigh and Collins, 2014). Therefore, regulation of NSC proliferation by PrP^C^ may be a consequence of its putative antioxidant properties or, conversely, protection from oxidative stress by PrP^C^ may result from a role in regulating physiological production of ROS for signalling purposes. PrP^C^ has also been observed to promote proliferation of colon adenocarcinoma cells (Chieng and Say, 2015), neuroblastoma cells (Llorens et al., 2013), cancer stem cells that give rise to glioblastoma (Corsaro et al., 2016), and precursor cells in intestinal organoid cultures (Besnier et al., 2015). In neuroblastoma cells, PrP^C^ was proposed to interact with the epidermal growth factor receptor to promote activation of the PI3K-Akt pathway (Llorens et al., 2013). PI3K-Akt-dependent mTOR activation might also drive the increased protein synthesis required for differentiation processes, such as changes to morphology (Roffe et al., 2010).

Although, PrP^C^-dependent regulation of neurite outgrowth, cell adhesion, expression of cytoskeletal proteins and proliferation could have been addressed separately, we argue that these putative functions can be understood in the context of PrP^C^ modulating different types of cellular differentiation. This explanation is attractive because, rather than affecting various cellular processes *via* independent mechanisms, PrP^C^ may, instead, regulate the function of a single interacting partner that underpins differentiation—given that altered regulation of cell adhesion would have an impact on many differentiation processes the interacting partner could be NCAM1, but there are many other possibilities and further research will be required to obtain a definitive answer.

### Neuronal excitability

As described in the previous section, interactions between PrP^C^ and mGLuRs have been linked to regulation of neurite outgrowth (Beraldo et al., 2011). There are also reports of PrP^C^ interacting with other neurotransmitter receptors, including α7 nicotinic acetylcholine (Beraldo et al., 2010), kainate (Carulla et al., 2011), α-amino-3-hydroxy-5-methyl-4-isoxazolepropionic acid (Kleene et al., 2007), and N-methyl-D-aspartate receptors (NMDARs) (Khosravani et al., 2008). NMDARs are a subclass of ionotropic glutamate receptors and are particularly implicated in excitotoxic neuronal cell death, which occurs when overactivation of NMDARs causes dysregulation of intracellular calcium homeostasis, leading to disruption of various physiological processes (Bondy and Lee, 1993). NMDAR-mediated excitotoxicity contributes to neuronal death in prion diseases and other neurodegenerative disorders (Muller et al., 1993; Gorman, 2008). Intriguingly, PrP^C^ has been reported to inhibit activity of NMDARs containing the GluN2D subunit, which suggests that PrP^C^ may protect neurons from excitotoxic death (Khosravani et al., 2008). There is evidence for this *in vivo*, for example, Zurich I PrP^C^-knockout mice displayed increased excitotoxicity in the retina in response to damaging light intensities (Frigg et al., 2006) and in the hippocampus following injection with N-methyl-D-aspartate (Khosravani et al., 2008), which selectively activates NMDARs. Given that excitotoxicity is the major cause of neuronal death following ischaemic stroke (Lai et al., 2014), it is also interesting to note that experimental stroke has been shown to induce PrP^C^ expression in the affected brain region in both rats and mice (Weise et al., 2004; Shyu et al., 2005). Furthermore, Zurich I PrP^C^-null mice displayed greater infarct volumes than wild type controls after transient or permanent middle cerebral artery occlusion (Spudich et al., 2005; Weise et al., 2006), whilst injection of a PrP^C^-expressing adenovirus construct into rat brains reduced infarct volume in a similar experimental model (Shyu et al., 2005). In addition to potentially modulating NMDAR activity, involvement of PrP^C^ in the cellular response to oxidative stress could explain the putative protective effect of PrP^C^ expression following stroke, since oxidative stress is a feature of excitotoxicity.

Increased sensitivity to seizures induced by high-dose kainate treatment has been observed in studies of Zurich I PrP^C^-null mice (Rangel et al., 2009; Carulla et al., 2011). However, this finding has been called into question by Striebel et al. (2013b), who presented evidence that *Prnp* flanking genes may be responsible for the seizure phenotype rather than *Prnp* itself. Further adding to the complexity is a recent report suggesting that, whilst *Prnp* flanking genes may affect sensitivity to kainate treatment, PrP^C^ expression also has a neuroprotective effect (Carulla et al., 2015). Additionally, one study found that hippocampal slices from Zurich I PrP^C^-null mice were actually more resistant than wild type controls to three different seizure-inducing protocols, including removal of Mg^2+^ from the culture medium, which leads to hyperexcitability of NMDARs, and treatment with gamma-aminobutyric acid (GABA) receptor antagonists (Ratte et al., 2011).

If PrP^C^ can modulate neuronal excitability then one might expect a lack of PrP^C^ expression to affect synaptic plasticity. Indeed, Collinge et al. (1994) showed that Zurich I PrP^C^-knockout mice had disrupted hippocampal long-term potentiation (LTP), a form of synaptic plasticity involved in memory formation. These mice displayed abnormal behaviour in nest building and novel environment exploration tasks and more pronounced age-related decline in short-term memory compared to wild type controls (Schmitz et al., 2014), effects that are possibly underpinned by impaired LTP. Intriguingly, it has been reported that the effect of ageing on memory can be blocked by infusing wild type mice with a peptide containing the putative PrP^C^-binding site of STI1 (Rial et al., 2009). Mice overexpressing PrP^C^ were similarly protected from age-related memory decline in the same study. NMDAR activity is required for LTP in the area of the hippocampus that was used by Collinge et al. (1994), suggesting that the disrupted LTP observed by the authors in PrP^C^-null hippocampal slices could have been caused by PrP^C^ ablation affecting NMDAR activity, as has been described previously (Khosravani et al., 2008). However, Collinge et al. (1994) found that the contribution of NMDAR-mediated currents to excitatory postsynaptic potentials was unaffected by PrP^C^ knockout. Instead, the lack of PrP^C^ expression resulted in weaker fast inhibitory postsynaptic potentials generated through GABA receptors. In contrast, a different study identified no effects of PrP^C^ knockout on inhibitory currents generated by GABA receptors nor, indeed, on LTP itself (Lledo et al., 1996).

Together, the findings reported in this section paint a confusing picture, indicating that further work is required to determine conclusively how PrP^C^ affects neuronal excitability. There is increasing recognition of the contribution of *Prnp* flanking genes to phenotypes displayed by PrP^C^-knockout mice that were generated on mixed genetic backgrounds, an issue that confounds interpretation of many studies connecting PrP^C^ to the regulation of neuronal excitability—this problem is documented in more detail in a couple of recent review articles (Striebel et al., 2013a; del Rio and Gavin, 2016). Nevertheless, there are data from animal models other than mice that support the involvement of PrP^C^ in the modulation of neuronal excitability. For example, knocking out PrP2 expression in zebrafish using zinc finger nuclease technology affected NMDAR currents and increased susceptibility to seizures induced by the drug pentylenetetrazol (Fleisch et al., 2013). Furthermore, in a study of the *Drosophila* neuromuscular junction, researchers found that murine PrP^C^ expression resulted in enlarged synaptic vesicles and increased the probability of neurotransmitter release (Robinson et al., 2014).

### Myelin maintenance

One phenotype of PrP^C^-knockout mice that was discovered relatively recently is a widespread, adult-onset demyelination of the PNS. This phenotype has now been observed in Zurich I and Npu knockout mice (Bremer et al., 2010) as well as the newly generated Zurich III line (Nuvolone et al., 2016), which provides strong evidence that PrP^C^ is involved in myelin maintenance. Intriguingly, Bremer et al. (2010) showed that neuron-specific PrP^C^ expression was sufficient to rescue the demyelination phenotype, whereas PrP^C^ expressed only in Schwann cells had little effect. More recent work has shown that PrP^C^ may promote myelin maintenance through an interaction between its extreme N-terminal region (residues 23–33) and G-protein coupled receptor 126 (GPR126) on the surface of Schwann cells (Kuffer et al., 2016). There is some evidence that α-cleavage of neuronal PrP^C^ is needed to prevent PNS demyelination (Bremer et al., 2010), perhaps indicating that the released N1 fragment, which contains the 23–33 region, is responsible for the interaction with GPR126. Binding of the PrP^C^ N-terminus seems to promote receptor activation, initiating signalling through the cyclic adenosine monophosphate (cAMP)-PKA pathway (Kuffer et al., 2016). In zebrafish, PKA acting downstream of GPR126 was shown to induce myelination during embryonic development by upregulating expression of E3 SUMO-protein ligase EGR2 (Glenn and Talbot, 2013), a transcription factor that also seems to regulate myelin maintenance (Decker et al., 2006). In spite of the importance of the GPR126-PKA signalling axis in the initial myelination of PNS axons, PrP^C^-null mice appear to develop morphologically-normal myelin before onset of the demyelinating polyneuropathy (Bremer et al., 2010). Kuffer et al. (2016) suggested that developmental myelination may progress normally in the PrP^C^-knockout mice because the lack of PrP^C^ expression is compensated for by other GPR126 ligands such as type IV collagen and laminin-211, which have both been reported to regulate GPR126 activity (Paavola et al., 2014; Petersen et al., 2015). However, it has also been reported that GPR126-PKA signalling is *not* required for myelin maintenance. For example, E3 SUMO-protein ligase EGR2 expression by zebrafish Schwann cells was shown to be independent of GPR126 signalling following completion of initial myelination (Glenn and Talbot, 2013). Thus, the molecular mechanisms by which PrP^C^ affects myelin integrity are still to be determined conclusively.

No myelin-related abnormalities have been identified within the CNS of PrP^C^-knockout mice (Bremer et al., 2010), perhaps because CNS myelination reportedly does not depend upon GPR126 activity (Monk et al., 2011). However, it is of note that spinal cord expression of PrP^C^ was found to be reduced in human patients with multiple sclerosis, a demyelinating disease of the CNS (Scalabrino et al., 2015).

### Circadian rhythm

The distinctive clinical feature of a human prion disease known as fatal familial insomnia is severe disruption of the sleep-wake cycle (Collins et al., 2001). Strikingly, PrP^C^-null mice also exhibit alterations to their sleep structure, including faster cycling through the different stages of sleep and more brief awakenings than wild type mice (Tobler et al., 1996, 1997; Sanchez-Alavez et al., 2007). These issues may be caused by changes to the regulation of melatonin production—melatonin is synthesised by the pineal gland and regulates sleep timing as well as other processes. Melatonin production is under the control of the suprachiasmatic nucleus of the hypothalamus, which is the master regulator of the circadian rhythms that drive the sleep-wake cycle. Thus, melatonin levels vary according to a 24-h cycle—for nocturnal animals such as mice, levels are highest in the hours of darkness and lower levels are required for sleep initiation. One study found that serum melatonin levels of Zurich I and Npu PrP^C^-knockout mice were considerably lower than in wild type mice during the dark phase of the cycle but were higher during the light phase (Brown et al., 2002), which probably contributed to the sleep disruption. A network of proteins called the circadian clock work together to generate circadian rhythms and, strikingly, *Prnp* mRNA levels seem to be subject to circadian oscillations in the suprachiasmatic nucleus in addition to other areas of the rat forebrain (Cagampang et al., 1999). Together, these lines of evidence suggest that PrP^C^ might have a regulatory role in the sleep-wake cycle, raising the possibility that a loss of this function is involved in the pathogenesis of fatal familial insomnia.

Given that metabolic processes are also subject to circadian regulation (Dibner et al., 2010), one might expect PrP^C^-knockout mice to show signs of altered metabolism. Interestingly, recent data suggest that PrP^C^ knockout does have an effect on glucose homeostasis. For example, Strom et al. (2011) demonstrated that, compared with wild type controls, blood glucose levels of PrP^C^-null mice were slower to return to normal after intraperitoneal injection of glucose. In the same study, pancreatic insulin secretion and the effects of intraperitoneal insulin injection on blood glucose levels were shown to be similar irrespective of PrP^C^ expression, however, it has been reported that insulin resistance develops more quickly in PrP^C^-null mice in response to a high fat diet (Brito et al., 2013). More work is required to unpick the mechanisms behind these phenotypes, although evidence obtained *in vitro* suggests that PrP^C^-mediated Fyn kinase signalling can lead to activation of hypoxia-inducible factor-2α and subsequent upregulation of glucose transporter 1 expression, resulting in increased glucose uptake by cells (Li et al., 2011). Additionally, glucose homeostasis is known to be regulated by the PI3K-Akt pathway (Schultze et al., 2012), which PrP^C^ has also been reported to modulate (Vassallo et al., 2005; Roffe et al., 2010; Llorens et al., 2013).

### Metal ion homeostasis

A number of early studies of PrP^C^ function focused on an apparent ability to bind Cu^2+^ ions at the cell membrane through the octapeptide repeat region (Hornshaw et al., 1995a,b; Brown et al., 1997a). Subsequently, PrP^C^ interactions with Cu^2+^ have been implicated in the regulation of NMDAR activity (Gasperini et al., 2015), astrocytic glutamate uptake (Brown and Mohn, 1999), protection against oxidative stress (Rachidi et al., 2003; Watt et al., 2007) and maintenance of Cu^2+^ homeostasis in the placenta (Alfaidy et al., 2013). In addition, binding of Cu^2+^ by PrP^C^ in the presence of ROS appears to promote β-cleavage of PrP^C^, leading to production of C2 and N2 (McMahon et al., 2001; Watt et al., 2005; McDonald et al., 2014). However, whilst it is clear that PrP^C^ can bind Cu^2+^ *in vitro*, the relevance of this interaction *in vivo* has been questioned. For example, it was reported that PrP^C^-dependent internalisation of Cu^2+^ by cells in culture occurred only when the extracellular Cu^2+^ concentration exceeded physiologically relevant levels (Rachidi et al., 2003). Furthermore, although some studies have shown that PrP^C^ expression can protect neuronal and non-neuronal cell lines from oxidative stress caused by excessive Cu^2+^ (Rachidi et al., 2003; Watt et al., 2007), Cingaram et al. (2015) found that transfecting PrP^C^ into the Zpl hippocampal cell line, originally derived from Zurich I PrP^C^-knockout mice, did not result in protection from Cu^2+^-mediated toxicity.

Experiments in neuronal cell lines showed that PrP^C^ transfection did not confer protection against the oxidative DNA damage and subsequent cell death caused by treatment with high levels of Mn^2+^, Co^2+^, or Zn^2+^ (Watt et al., 2007; Cingaram et al., 2015). However, PrP^C^ expression did protect neuroblastoma cells from the toxic effects of Fe^2+^ treatment (Watt et al., 2007). Furthermore, PrP^C^ appears to be involved in the physiological uptake of iron by cells; this was first shown in neuroblastoma cells, which became more efficient at sequestering iron from the culture medium following overexpression of PrP^C^ (Singh et al., 2009b). Singh et al. (2009a) also showed that PrP^C^ transfection increased expression of the iron-storage protein ferritin and reduced expression of both the iron-transport protein transferrin and the transferrin receptor. These alterations to the expression levels of iron-binding proteins are part of the homeostatic response that ensures that the amount of labile iron within cells remains constant when iron uptake is enhanced (Hare et al., 2013). Further evidence supporting a role for PrP^C^ in iron homeostasis has been obtained *in vivo*, where the liver, spleen, brain and kidneys of Zurich I PrP^C^-null mice have all been shown to have lower total levels of iron than wild type controls (Singh et al., 2009a; Haldar et al., 2015). Consequently, PrP^C^-knockout also reduced ferritin expression and increased expression of transferrin and its receptor in various tissues (Singh et al., 2009a). Most iron circulating in the bloodstream is in the form of Fe^3+^ bound to transferrin (Hare et al., 2013); however, Fe^3+^ must be reduced to Fe^2+^ by a ferrireductase to enable uptake by cells. PrP^C^ itself may have ferrireductase activity (Singh et al., 2013; Haldar et al., 2015) and may act to enhance iron uptake through divalent-metal transporter 1 and ZIP14 (Tripathi et al., 2015), although an alternative explanation is that PrP^C^ binds through its octapeptide repeat region to a ferrireductase in order to modulate its activity (Tripathi et al., 2015). One final point to make is that PrP^C^-null mice are only mildly anaemic, probably because, in spite of the low levels of stored iron, sufficient quantities of labile iron were present to maintain normal cellular function (Singh et al., 2009a).

In conclusion, more than two decades after the discovery that PrP^C^ can bind Cu^2+^, the functional significance of this interaction is still unclear. However, the recent reports proposing that PrP^C^ is involved in iron homeostasis are intriguing. Further research is necessary to clarify the mechanisms responsible and to determine whether other PrP^C^-knockout mouse lines also display signs of anaemia.

### Roles in the immune system

Because prion diseases are neurodegenerative disorders, much of the research into PrP^C^ function has focused on its role in the nervous system. However, PrP^C^ is also expressed in immune cells, including T-lymphocytes, natural killer cells, macrophages and mast cells (Durig et al., 2000; Haddon et al., 2009). Mast cells reportedly express high levels of PrP^C^, some of which is shed from the cell membrane as the N3 fragment (Haddon et al., 2009). When activated, mast cells release various inflammatory mediators, including histamine, prostaglandins and cytokines (Stempelj and Ferjan, 2005). Interestingly, treatment of cultured mast cells with compounds that induce activation resulted in rapid shedding of a large proportion of the cellular pool of PrP^C^, suggesting that PrP^C^ could be involved in the inflammatory response (Haddon et al., 2009). Mast cells are also involved in angiogenesis (Maltby et al., 2009), a process that PrP^C^ seems to modulate within the placenta (Alfaidy et al., 2013), although this function may be more linked to the putative antioxidant properties of PrP^C^. Moreover, mast cells release pain mediators upon activation (Chatterjea and Martinov, 2015) and ablation of PrP^C^ expression in mice is associated with altered sensitivity to pain. However, whilst one study found that PrP^C^-null mice showed a hypersensitivity to pain (Gadotti and Zamponi, 2011), another demonstrated that PrP^C^-null mice were more resistant than wild type controls (Meotti et al., 2007). It is conceivable that the apparent influence of PrP^C^ expression on pain sensitivity stems from a role in regulating mast cell function, although Gadotti and Zamponi (2011) provided evidence that loss of PrP^C^-dependent NMDAR inhibition was responsible for the hypersensitivity to pain displayed by the PrP^C^-knockout mice in their study.

PrP^C^ expression by T-cells may be involved in their differentiation, since knockdown of PrP^C^ resulted in an increased tendency for T-cells to develop a pro-inflammatory phenotype (Hu et al., 2010). Additionally, in a model of autoimmune encephalomyelitis, PrP^C^-knockout mice experienced a more severe disease phenotype that was linked to increased T-cell-dependent neuroinflammation (Tsutsui et al., 2008), possibly because PrP^C^ is involved in the signalling complexes that regulate T-cell activation (Mabbott et al., 1997; Mattei et al., 2004). In contrast, another study employing the autoimmune encephalomyelitis model reported no evidence that T-cells were driven toward a more inflammatory phenotype by PrP^C^ knockout and, instead, the increased disease severity seemed to result from loss of PrP^C^ expression within the CNS, not the immune system (Gourdain et al., 2012).

As explained previously, the first non-laboratory animals lacking PrP^C^ expression were recently identified (Benestad et al., 2012). No overt abnormalities were detected in these PrP^C^-null goats (Benestad et al., 2012), although increased neutrophil numbers compared with PrP^C^-expressing controls were reported recently (Reiten et al., 2015). Further study of these PrP^C^-null goats may help to improve understanding of how PrP^C^ expression influences the immune system, knowledge that is currently rather limited. Of particular interest is the putative function of PrP^C^ expression in mast cells, given the multiple lines of circumstantial evidence, detailed in this section, that hint at an important role for PrP^C^ in these cells.

### Mitochondrial homeostasis

Another function sometimes ascribed to PrP^C^ is maintenance of mitochondrial homeostasis. For instance, whilst mouse studies have shown that PrP^C^-knockout does not affect the membrane potential or baseline respiration rate of mitochondria, nor the activities of the individual complexes of the electron transport chain (Brown et al., 2002; Miele et al., 2002; Lobao-Soares et al., 2005), transcriptomic, and proteomic analyses of brain tissue and cultured cells have identified specific subunits of each of the five electron transport chain complexes that vary in their expression between PrP^C^-null and wild type mice (Miele et al., 2002; Ramljak et al., 2008; Stella et al., 2012). Furthermore, the Npu line of PrP^C^-null mice were found to have reduced numbers of mitochondria in the brain and myocardium, although the mitochondria that were present were larger and had increased maximal respiratory capacities, presumably to compensate for the lower numbers (Miele et al., 2002; Paterson et al., 2008). The enhanced maximal respiratory capacity of individual PrP^C^-null mitochondria seemed to be enabled by increased activity of electron transport chain complex I, which was, in turn, associated with an increase in superoxide production (Paterson et al., 2008). In line with these findings, superoxide dismutase 2, which is found within mitochondria and converts superoxide into either hydrogen peroxide or molecular oxygen, was reportedly more active in PrP^C^-null mice (Brown et al., 1997b; Miele et al., 2002; Paterson et al., 2008). Furthermore, it is conceivable that the reported antioxidant properties of PrP^C^ could derive from a role in maintaining mitochondrial homeostasis and, consequently, preventing excessive production of ROS. It should be emphasised, however, that the evidence suggesting that PrP^C^ may regulate mitochondrial function comes from a very limited number of studies and, as far as we aware, the only published examples of mitochondrial phenotypes in PrP^C^-null mice are from studies of the Npu line.

### Roles in regulating levels of amyloid beta and tau

β-Cleavage of the amyloid precursor protein (APP) by beta-secretase 1 produces a fragment called sAPPβ that is subsequently cleaved by gamma-secretase to produce amyloid beta (Aβ) peptides. Aβ accumulates during pathogenesis of Alzheimer's disease (AD) and PrP^C^ reportedly acts as an inhibitor of β-secretase 1 (Parkin et al., 2007; Whitehouse et al., 2013), thereby reducing the amount of Aβ produced and suggesting that PrP^C^ expression should protect against the development of AD. A feedback loop has also been proposed that consists of the APP intracellular domain (the other fragment produced by cleavage of sAPPβ) activating cellular tumour antigen p53, which subsequently upregulates PrP^C^ expression, leading to further inhibition of β-secretase 1 activity (Vincent et al., 2009). However, recent data have questioned whether the APP intracellular domain is able to induce PrP^C^ expression (Lewis et al., 2012), whilst another study found that PrP^C^ expression actually led to higher rates of APP β-cleavage (McHugh et al., 2012). In addition, removing PrP^C^ from human APP-expressing mice has no effect on processing or Aβ levels (Whitehouse et al., 2016).

In addition to potentially affecting APP processing, it has been suggested that PrP^C^ downregulates transcription of microtubule-associated protein tau by activating Fyn kinase signalling (Chen et al., 2013). This putative function would also be protective against AD, since accumulation of neurofibrillary tangles consisting of hyperphosphorylated tau is another major molecular feature of the disease. Conversely, oligomeric forms of Aβ are reported to bind to PrP^C^ at either its extreme N-terminal region (Chen et al., 2010; Dohler et al., 2014) or between residues 92 and 110 (Lauren et al., 2009), potentially eliciting toxic effects through hyperactivation of Fyn kinase (Vergara et al., 2015). The C1 fragment, created by α-cleavage of PrP^C^, lacks both the putative Aβ binding sites. Therefore, the reduced levels of hippocampal PrP^C^ and the increased rates of PrP^C^ α-cleavage in the cortex that have been reported in the brains of sporadic AD patients could be part of a protective response (Whitehouse et al., 2010; Beland et al., 2014). On the other hand, one study found that higher levels of PrP^C^ in serum were correlated with poorer cognitive function in an elderly human population, although the increase in serum PrP^C^ could be a consequence of loss of PrP^C^ from neuronal membranes (Breitling et al., 2012). Overall, the conflicting nature of the evidence obtained to date makes it difficult to determine whether the net effect of PrP^C^ expression on AD pathogenesis is positive or negative.

### Interacting partners of PrP^C^

The preceding sections of this review have addressed many of the diverse functions ascribed to PrP^C^. Putative interacting partners of PrP^C^ that may be involved in these functions have also been mentioned and Table 2 provides information about some of the interactions best supported by the literature. Given that the GPI-anchored PrP^C^ molecule lacks an intracellular domain, it is likely that the protein interacts with one or more co-receptors at the cell surface to mediate downstream signalling. Several potential co-receptors are included in Table 2, such as NCAM1 and mGluRs, which may be involved in PrP^C^-dependent neurite outgrowth (Santuccione et al., 2005; Beraldo et al., 2011). Furthermore, the extracellular matrix protein laminin may act as a PrP^C^ ligand (Graner et al., 2000a) to activate neuritogenic signalling downstream of mGluRs (Beraldo et al., 2011). Likewise, STI1 released by astrocytes (Lima et al., 2007) may form a complex with nicotinic acetylcholine receptors and PrP^C^ on the neuronal surface, leading to activation of the acetylcholine receptors and consequent pro-survival and neuritogenic signalling (Beraldo et al., 2010). Interactions between PrP^C^ and neurotransmitter receptors are also implicated in the regulation of neuronal excitability, although some of these findings have been disputed, as explained previously. Further PrP^C^ binding partners listed in Table 2 include proteins that are important at cell-cell junctions, such as desmoplakin and junction plakoglobin (Zafar et al., 2011; Besnier et al., 2015), and may be linked to the role PrP^C^ is thought to play in the regulation of cell adhesion (Malaga-Trillo et al., 2009; Sempou et al., 2016). Additionally, PrP^C^ reportedly binds to cytoskeletal proteins, such as tubulin and vimentin (Nieznanski et al., 2005; Zafar et al., 2011), although, given that PrP^C^ is usually expressed at the cell surface, these findings could be artefactual. For example, cell or tissue lysis carried out prior to analysis of protein-protein interactions can result in association of proteins that do not interact physiologically because of their differing subcellular localisation. An alternative explanation is that PrP^C^ does interact with cytoskeletal proteins but does so indirectly—not all techniques used to identify binding partners of a protein can distinguish between direct and indirect interactions. Desmoplakin, for example, could provide the link between PrP^C^ and tubulin or vimentin, since desmoplakin is reported to interact with the microtubule-binding protein end-binding 1 (Patel et al., 2014) and with vimentin intermediate filaments (Stappenbeck and Green, 1992). In fact, many of the proteins that appear to interact with PrP^C^ may merely be part of the same multiprotein complex or complexes rather than being direct binding partners. Alternatively, the relatively flexible structure of the PrP^C^ N-terminal domain may allow the protein to bind directly to multiple partners (Bakkebo et al., 2015), potentially enabling PrP^C^ to act as a scaffolding protein that mediates the formation of a number of different multiprotein complexes at the cell surface, as has been proposed previously (Linden et al., 2008).

**Table 2:** **Reported interacting partners of PrP^C^**.

**Protein/protein complex**	**Subcellular localisation and function**	**Method**	**References**
14-3-3 protein	Cytoplasmic adaptor protein involved in multiple signalling pathways	Interactomics; co-IP	Satoh et al., 2005; Zafar et al., 2011
37/67 kDa laminin receptor	Cell surface receptor for laminin (see below)	Binding assay; yeast two-hybrid; co-IP	Gauczynski et al., 2001; Hundt et al., 2001
60 kDa heat shock protein	Mitochondrial chaperone	Interactomics; co-IP	Satoh et al., 2005; Zafar et al., 2011
Annexin A2	Calcium-regulated cell membrane protein with a poorly defined function	Interactomics; co-IP	Morel et al., 2008; Zafar et al., 2011
Desmoplakin	Organisation of cell junctions	Interactomics; co-IP	Morel et al., 2008; Besnier et al., 2015
Dipeptidyl aminopeptidase-like protein 6	Cell membrane protein that binds to and modulates activity of potassium channels	Interactomics; co-IP	Schmitt-Ulms et al., 2004; Mercer et al., 2013
Doppel	Cell membrane protein of unknown function	Co-IP	Qin et al., 2006; Caputo et al., 2010
Junction plakoglobin (a.k.a. γ-catenin)	Organisation of cell junctions	Interactomics; yeast two-hybrid	Besnier et al., 2015; Lappas Gimenez et al., 2015
Laminin	Extracellular matrix protein with multiple functions (cell migration, adhesion, differentiation…)	Binding assay	Graner et al., 2000a; Coitinho et al., 2006
Lactate dehydrogenase	Cytoplasmic enzyme that converts lactate to pyruvate and *vice versa*	Interactomics; co-IP	Zafar et al., 2011; Ramljak et al., 2015
Metabotropic glutamate receptor	Cell surface receptor for the neurotransmitter glutamate	Co-IP; binding assay	Beraldo et al., 2011; Haas et al., 2014
Neural cell adhesion molecule 1	Cell membrane protein with multiple functions (adhesion, neurite outgrowth…)	Co-IP; binding assay	Schmitt-Ulms et al., 2001; Santuccione et al., 2005
Nicotinic acetylcholine receptor	Cell surface receptor for the neurotransmitter acetylcholine	Co-IP; binding assay	Petrakis et al., 2008; Beraldo et al., 2010
Stress-induced phosphoprotein 1	Cytoplasmic co-chaperone; may also be secreted to function as a PrP^C^ ligand	Binding assay; co-IP	Zanata et al., 2002; Lopes et al., 2005
Tubulin	Cytoskeletal protein (microtubules)	Interactomics; co-IP	Nieznanski et al., 2005; Zafar et al., 2011
Vimentin	Cytoskeletal protein (intermediate filaments)	Interactomics; yeast two-hybrid	Zafar et al., 2011; Lappas Gimenez et al., 2015

*Some of the proteins that may interact with PrP^C^. Numerous interacting partners have been suggested but we include only those proteins for which two separate references could be found that provided direct evidence of an interaction with PrP^C^. This is not meant to indicate that interactions identified in a single reference are incorrect, especially in the case of recently identified interactions. It is also important to state that certain techniques, such as co-immunoprecipitation (co-IP), do not distinguish between indirect and direct interactions. Therefore, some of the proteins listed in this table may interact with PrP^C^ indirectly*.

### Signalling pathways affected by PrP^C^ expression

**Figure 5:**
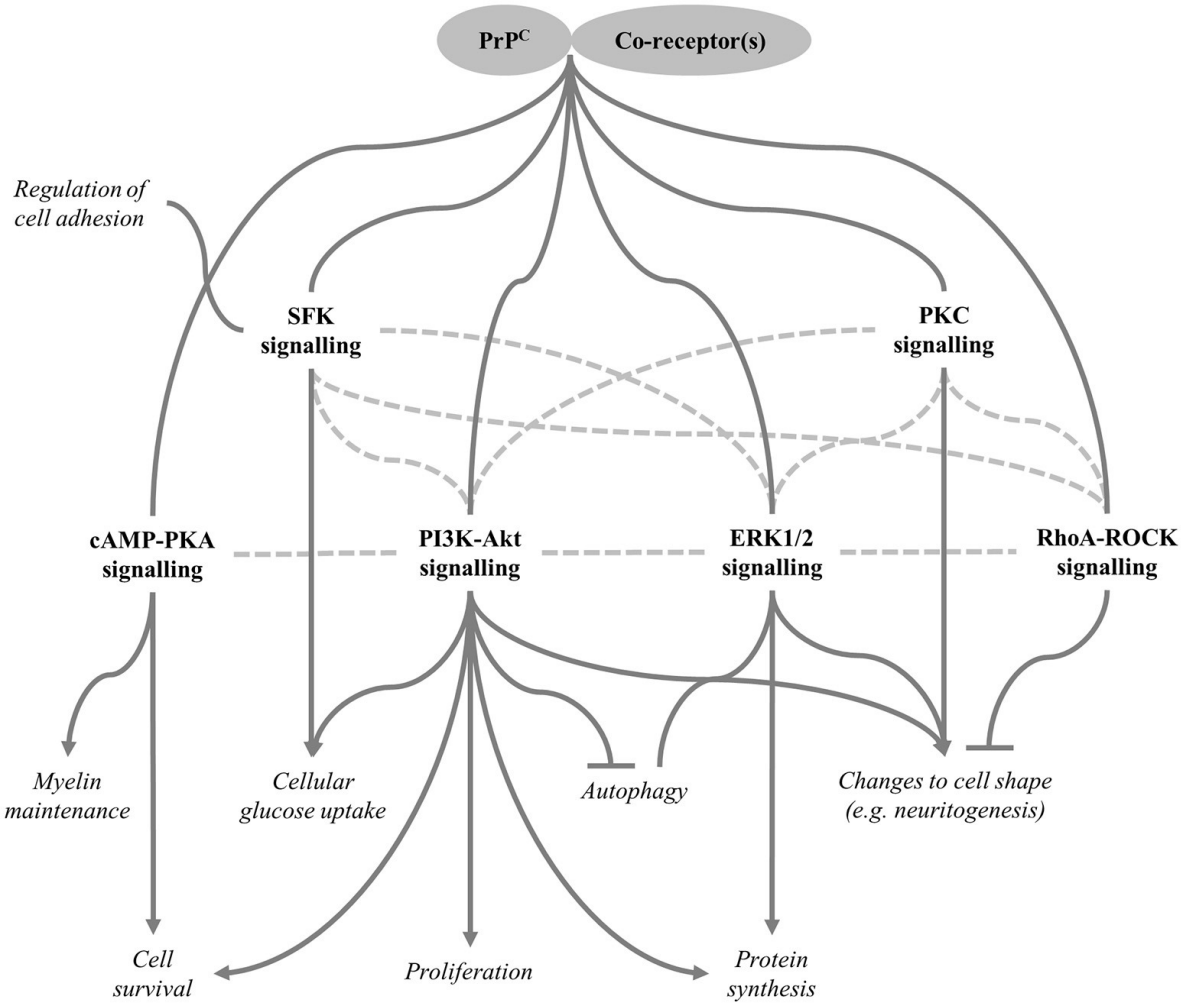
**Signalling pathways regulated by PrP^C^**. Various downstream signalling pathways are reportedly modulated as a result of PrP^C^ interacting with specific co-receptors (some candidate co-receptors are included in Table 2). Cellular functions regulated by these pathways are shown in italics. Arrows indicate positive regulation, inhibition is shown by the flat-ended lines, and connectors without arrows indicate that the direction of regulation may be context-specific. Dotted lines represent crosstalk between pathways; therefore, some of the pathways shown may be regulated indirectly through other pathways rather than modulated directly by PrP^C^. SFK, Src family kinase, which includes Fyn kinase.

Interactions between PrP^C^ and its co-receptor(s) initiate downstream signalling and Figure 5 provides an overview of the signalling pathways that seem to be affected by PrP^C^ expression as well as some of the cellular processes that may be regulated in a PrP^C^-dependent manner through these pathways. Further information on these signal transduction pathways was retrieved from several review articles (Dhillon et al., 2007; Newton, 2010; Rosse et al., 2010; Zhang and Yu, 2012; Martini et al., 2014), enabling some of the crosstalk between pathways to be included in the figure. In brief, evidence suggests that PrP^C^ expression affects the activities of the ERK1/2 (Lopes et al., 2005; Caetano et al., 2008; Beraldo et al., 2011) and PI3K-Akt signalling pathways (Vassallo et al., 2005; Roffe et al., 2010; Llorens et al., 2013), both of which are involved in functions ascribed to PrP^C^ such as regulation of protein synthesis (Roffe et al., 2010) and autophagy (Nah et al., 2013; Shin et al., 2014). In addition, differential activation of the PI3K-Akt pathway may explain the effects of PrP^C^ expression on proliferation (Llorens et al., 2013). Furthermore, PrP^C^ appears to modulate cAMP-PKA signalling to stimulate the maintenance of PNS myelin (Kuffer et al., 2016) and to promote cell survival (Lopes et al., 2005), although it should be stressed that the literature as a whole does not provide strong support for PrP^C^ having a direct pro-survival role, as explained previously. PrP^C^ expression also seems to activate signalling through Src family kinases, such as Fyn kinase (Krebs et al., 2007; Chen et al., 2013), leading to changes in cell adhesion properties (Sempou et al., 2016) as well as potentially affecting the cellular uptake of glucose (Li et al., 2011), a process that can additionally be regulated by the PI3K-Akt pathway (Schultze et al., 2012). Finally, PrP^C^-dependent regulation of several pathways, including RhoA-ROCK (Loubet et al., 2012) and PKC (Beraldo et al., 2011) signalling, reportedly results in changes to cell morphology, such as neuritogenesis.

The extensive nature of the crosstalk between pathways affected by PrP^C^ expression raises the possibility that they may not all be regulated by PrP^C^ directly; instead, the altered activation states of some of the pathways may be indirect consequences of effects on other signalling cascades. Moreover, although PrP^C^ expression has an activating effect on most of the pathways shown in Figure 5, it is highly unlikely that this is the case for all tissue/cell types. For example, the PI3K-Akt pathway is thought to promote proliferation (Martini et al., 2014) and yet PrP^C^ expression has been shown to have opposing effects on proliferation in different experimental models. Context-dependent regulation of cell signalling processes by PrP^C^ could result from co-receptors and/or downstream components of the relevant signalling pathways being differentially expressed in different tissue/cell types.

## Final conclusions and future perspectives

Since it was first recognised that prion protein misfolding plays an important role in the pathogenesis of prion diseases, significant attention has been directed toward understanding the physiological function of PrP^C^. One goal of such research has been to determine whether the disease processes of prion diseases are driven mainly by toxic gain of function caused by conversion of PrP^C^ into PrP^Sc^ or by a loss of normal PrP^C^ function. However, this question has yet to be answered conclusively, because a clear description of PrP^C^ function remains elusive. This is not to say that no progress has been made; in fact, PrP^C^ has been linked to roles in a wide variety of cellular processes. Furthermore, numerous putative interacting partners of PrP^C^ have been identified and PrP^C^ expression has been shown to affect a number of downstream signalling pathways. These findings have led to proposals that PrP^C^ is a scaffolding protein that regulates the formation of various multiprotein complexes at the cell surface. However, given the potential for experimental artefacts when studying protein-protein interactions in addition to the possible detection of indirect rather than direct PrP^C^ binding partners, the actual number of direct PrP^C^ interactors may be considerably lower than it appears. It is also possible that some of the signalling processes reportedly affected by PrP^C^ expression are differentially activated due to crosstalk with other pathways rather than as a result of direct regulation by PrP^C^. Further adding to the complexity of understanding PrP^C^ function is recent evidence that has weakened support for the involvement of PrP^C^ in processes such as stress-protection, copper homeostasis and the modulation of neuronal excitability.

Arguably, the best-supported phenotype of PrP^C^-knockout mice is adult-onset demyelination of the PNS, suggesting that PrP^C^ expression is important for myelin maintenance. Additionally, there is accumulating evidence that PrP^C^ expression modulates proliferation, differentiation, adhesion properties and cell shape in multiple cell types. Given that proliferation rate, adhesion properties and cell shape are all likely to be affected when cells differentiate, we have suggested in this review that PrP^C^ may regulate a single underlying mechanism connected to differentiation and further work will be required to determine whether or not this is the case. Another intriguing phenotype of PrP^C^-null mice is altered sleep structure, which may result from disruption of the normal circadian variation in melatonin levels. However, there have been few publications on this topic, which is surprising since a role of PrP^C^ in sleep regulation would suggest that loss or subversion of normal PrP^C^ function could be involved in the development of fatal familial insomnia. Other areas of study that could be explored further include the link between PrP^C^ and iron homeostasis as well as the putative involvement of PrP^C^ in immune function, especially in the context of mast cells, which reportedly shed large quantities of the N3 fragment from the cell surface when activated.

Aside from the involvement of prion protein in prion diseases, there are wider reasons for pursuing investigations to clarify the function of PrP^C^. Firstly, given that PrP^C^ has been shown to affect processes such as proliferation, cellular differentiation and adhesion, the expression levels of PrP^C^ are likely to have an influence on cancer progression. Although, some attention has been focused on this topic in recent years, further research would help to determine whether treatments that modulate PrP^C^ activity or expression levels are beneficial in cancer. Secondly, the putative connection between PrP^C^ and glucose homeostasis makes the protein a potential target for treating metabolic disorders such as diabetes. Thirdly, given that dementia is one of the most pressing medical issues of our time, the apparent role of PrP^C^ in the cell biology of AD warrants further attention, especially since the relevant data obtained to date are rather contradictory. Together, these areas provide further impetus for continued research into the cell biological mechanisms regulated by PrP^C^, raising the possibility that a clear understanding of PrP^C^ function will be obtained in the near future.

## Author contributions

AC conducted the literature review and wrote the first draft of the manuscript. AG supervised the work, prepared publication-ready figures and corrected the manuscript ready for publication.

### Conflict of interest statement

The authors declare that the research was conducted in the absence of any commercial or financial relationships that could be construed as a potential conflict of interest.
